# Congenital Parvovirus B19 During the 2024 European Resurgence: A Prospective Single-Centre Cohort Study

**DOI:** 10.3390/pathogens14080798

**Published:** 2025-08-09

**Authors:** Pasqua Betta, Roberta Leonardi, Carmine Mattia, Alessandro Saporito, Silvia Gentile, Laura Trovato, Concetta Ilenia Palermo, Guido Scalia

**Affiliations:** 1 Neonatal Intensive Care Unit, University Hospital Policlinico “G. Rodolico San Marco”, University of Catania, 95123 Catania, Italy; lorettamattia@hotmail.com (C.M.); alessandrosaporito@hotmail.com (A.S.); 2 Postgraduate Training Programme in Pediatrics, Department of Clinical and Esperimental Medicine, University of Catania, 95123 Catania, Italy; 3 U.O.C. Laboratory Analysis Unit, A.O.U. ‘Policlinico-Vittorio Emanuele’, University of Catania, 95125 Catania, Italy; silvia.gentile7@gmail.com (S.G.); ltrovato@unict.it (L.T.); palermoilenia77@gmail.com (C.I.P.); lido@unict.it (G.S.); 4 Department of Biomedical and Biotechnological Sciences, University of Catania, 95123 Catania, Italy

**Keywords:** Parvovirus B19 congenital infection, neonate, Neonatal Intensive Care Unit (NICU), neonatal infection, troponin, cardiac biomarkers, biomarker trend, vertical transmission, B19V outbreak

## Abstract

Parvovirus B19 (B19V) re-emerged across Europe in 2024, raising concerns about vertical transmission and neonatal morbidity. We undertook a prospective, single-centre cohort study to characterise the early clinical course of congenitally infected neonates born between April and December 2024. Seventy-one pregnancies with serologically or PCR-confirmed maternal infection were enrolled; seven neonates met laboratory criteria for congenital B19V infection and were followed with serial clinical, biochemical and imaging assessments through the first year of life. Troponin I and CK-MB were measured on days 1, 3, 7 and 15; electrocardiogram (ECG) and echocardiography were repeated in parallel, and cranial ultrasound (US), ophthalmologic and audiologic screening were scheduled prospectively. Mean troponin rose from 50.7 ng L^−1^ on day 1 to a peak of 120.7 ng L^−1^ on day 7 (*p* < 0.01), normalising by one month, while echocardiograms remained structurally normal, and only one transient arrhythmia was recorded. CK-MB exceeded the reference range in 29% of infants but showed no clinical sequelae. Multiple periventricular hyperechogenicities were identified in 8/70 neonates (11%), and moderate anaemia (Hb ≤ 9.8 g/dL) occurred in 2 cases. Serum PCR detected high-level viraemia (>10^8^ genome equivalents mL^−1^) in 40% of those tested; saliva and urine were consistently negative. No instances of myocarditis or hydrops were observed. Our findings indicate that congenital B19V infection during the current outbreak is marked by transient biochemical myocardial stress and subtle neurosonographic changes rather than overt cardiac disease, supporting an outpatient-focused follow-up strategy incorporating serial biomarkers and targeted neuroimaging.

## 1. Introduction

Parvovirus B19 (B19V) is a ubiquitous, globally endemic, non-enveloped parvovirus with a linear single-stranded DNA genome of 5596 nucleotides, including inverted terminal repeats [1,2]. While it is classically associated with erythema infectiosum (fifth disease) in children—a typically mild or asymptomatic condition—its implications during pregnancy are far more severe [3].

Since early 2024, several European countries have seen an increase in B19V infections, especially in pregnant women and children, highlighting the need for enhanced epidemiological surveillance due to limited routine monitoring and the potential impacts of COVID-19-related immunity gaps [4,5,6,7].

A significant proportion of women of childbearing age (34–65%) enter pregnancy without immunity, and during epidemic periods, seroconversion rates can reach up to 13% [8]. Vertical transmission occurs in approximately 30–39% of maternal infections [9,10], particularly when maternal infection occurs within the first 20 weeks of gestation, thereby predisposing the foetus to serious complications [8].

Foetal infection with B19V can result in severe anaemia and hydrops fetalis due to the virus’s predilection for erythroid progenitors [11]. In addition, although less common, there is emerging evidence linking B19V to brain anomalies and long-term neurodevelopmental issues [8,9].

Accurate diagnosis of both maternal and foetal infection is critical. Maternal infection is typically confirmed via serologic assays for IgG and IgM antibodies, while foetal infection is established using polymerase chain reaction (PCR) testing. The combined detection of B19V-specific IgM and viral DNA yields the highest sensitivity [10,12]. In clinical practice, management of foetal complications primarily involves monitoring for anaemia through Doppler ultrasound (US) assessments of the middle cerebral artery, with intrauterine blood transfusion reserved for severe cases [13].

Beyond these hematologic and neurodevelopmental consequences, recent observations suggest that B19V may also compromise foetal myocardial integrity [14].

Wang et al. found B19V DNA in the cardiac tissues of 29 children born to infected mothers, with foetal myocarditis, monitored via US parameters, correlating with second-trimester maternal infection, yet no post-natal myocarditis has been reported; while B19V is linked to myocarditis, cardiomyopathy, and Kawasaki disease, and its nuclear localization in some congenital heart-diseased tissues hints at a possible connection with congenital heart disease, definitive follow-up guidelines and evidence remain lacking [15].

Despite the utility of echocardiography and electrocardiography (ECG) in evaluating cardiac function, these modalities may fail to detect subtle myocardial injury. In this context, sensitive biomarkers, particularly cardiac troponin, offer a promising alternative for identifying early myocardial involvement [16,17].

Given the recent surge in B19V cases across Europe and the lack of routine prenatal screening, we developed a comprehensive, evidence-based monitoring protocol. The aim of this prospective study was to evaluate virological, haematological, and cardiac parameters, particularly troponin, thereby enhancing neonatal risk stratification and clinical management strategies to improve perinatal outcomes.

## 2. Materials and Methods

### 2.1. Study Design

This is a prospective observational cohort study designed to evaluate the prognostic and diagnostic implications, including myocardial involvement in neonates with congenital B19V infection. This monocentric study was conducted at the Neonatal Intensive Care Unit (NICU) of the Policlinico G. Rodolico in Catania, Italy, from April to December 2024.

### 2.2. Study Population

A total of 71 pregnancies with confirmed maternal B19V infection were prospectively enrolled. Maternal infection or suspicion of infection was determined with the positivity of IgG and IgM antibodies or only IgM positivity, respectively, using serologic assays; the infection was subsequently confirmed through polymerase chain reaction (PCR) testing for B19V DNA in serum samples. Newborns of mothers infected were included in the study. In all enrolled cases, congenital B19V infection at birth was assessed through molecular testing in serum and/or IgM positive. All cases demonstrated normal cardiorespiratory adaptation immediately following delivery. Our inclusion criteria were as follows: positivity to IgM and/or IgG and/or positivity to serum PCR to B19V and, for the admission in NICU, the presence of symptoms.

### 2.3. Data Collection and Measurements

Upon delivery, a standardised clinical and diagnostic protocol was implemented for each newborn.

This baseline evaluation included the following:

Virological PCR Testing: Serum samples were analysed for the presence of B19V DNA using a quantitative PCR test (B19V ELITe MGB Kit; ELITechGroup, Torino, Italy), following the manufacturer’s instructions (limit of detection (LoD) of 250 copy/mL).

Serologic Testing: A chemiluminescent immunoassay (CLIA) for the quantitative determination of specific IgG and IgM antibodies to B19V was used to ascertain the timing and nature of the exposure. Assay (from DiaSorin, Saluggia, Italy) formats varied: in 2018–2023, LIAISON Biotrin Parvovirus B19 IgG/IgM, Index Value in the range 0–46 for IgG and 0–48 for IgM; from 2024, LIAISON Biotrin Parvovirus B19 IgG/IgM plus, results as IU/mL in the range 0–150 for IgG and as Index Values in the range 0–48 for IgM. Maternal acute infection was defined by IgM positivity and/or detection of B19V DNA, alternatively by contemporary positivity to IgM and IgG; isolated IgG positivity was interpreted as past exposure only. Congenital infection required neonatal virological confirmation (serum B19V DNA by real-time PCR within 48 h of life) eventually associated with neonatal B19V-IgM.

Haematological and Biochemical Evaluation: A complete blood count was performed. Biochemical assessments included liver and renal function tests.

Cardiac Assessment: Serum levels of cardiac troponin and creatine phosphokinase myocardial band (CK-MB) were measured as indicators of myocardial involvement. A 12-lead electrocardiogram (ECG) and an echocardiogram were conducted to assess cardiac structure and function.

Additional Evaluations: Cranial ultrasound (via the anterior fontanelle) was performed. Ophthalmologic and audiologic examinations were initiated to detect any early multisystem involvement.

Additionally, to systematically document the evolution of both virological and clinical parameters, serial evaluations were conducted at predetermined time points:

Day 1 (Admission): Comprehensive baseline evaluation, as detailed above.

Day 3, Day 7, and Day 15: Repeat assessments of B19V PCR, serology, complete blood count, and cardiac biomarkers (troponin and CK-MB) were performed. In addition, ECG and echocardiography were repeated as needed to monitor any dynamic changes in cardiac function.

In addition, an extended follow-up was performed:

At 1 Month: A follow-up visit was scheduled for repeat ECG and echocardiography to monitor ongoing cardiac function.

At 3 Months: Laboratory re-evaluation including a complete blood count and PCR for B19V DNA was conducted.

At 9 Months: A comprehensive re-assessment was performed, including repeat serologic testing (IgG and IgM) and PCR for B19V DNA in serum, saliva, and urine.

At 3, 6, 9, and 12 Months: Serial ophthalmologic evaluations, cranial ultrasounds, and neuro-paediatric assessments were scheduled to monitor for any emerging sequelae.

This study reported preliminary data collected during the first month of life in the enrolled neonates. Future directions include the continuation of clinical follow-up with a specific focus on both neurological and cardiac development. Neurological outcomes will be assessed using the Hammersmith Neonatal Neurological Examination and the Bayley Scales of Infant and Toddler Development, Third Edition (Bayley-III). Importantly, no patients have been lost to follow-up, and the data presented here reflect a complete dataset for the neonatal period.

Cardiac biomarkers were measured until normalization was confirmed. Finally, otoacoustic emissions were obtained at the earliest possible time to evaluate auditory function.

### 2.4. Objectives and Outcomes

Our primary outcome was to analyse serial measurements of serum troponin and CK-MB levels as biomarkers for myocardial involvement. The temporal trend in troponin levels was used to assess the severity and resolution of any myocardial injury. Troponin and CK-MB dosage was assessed on the day of admission, then on the third, seventh and fifteenth days of life.

Regarding our secondary outcomes, we evaluated ECG and echocardiography as indicators of cardiac function. Additionally, we evaluated haematologic, inflammatory and virological markers. Finally, neurological, ophthalmologic, and audiologic outcomes were determined at the scheduled follow-up assessments.

### 2.5. Statistical Analysis

Data were analysed using appropriate descriptive and inferential statistical methods using the MedCalc statistical software version 17.9.2 (MedCalc Software bvba, Ostend, Belgium; http://www.medcalc.org; 2017; accessed on 25 March 2025). Continuous variables (e.g., troponin levels, CK-MB) are presented as means ± standard deviations, and categorical variables are expressed as frequencies and percentages. Serial measurements were analysed using repeated measures analysis of variance (ANOVA) to evaluate trends over time, with post hoc comparisons as appropriate. Categorical variables are reported as percentages and compared using the two-tailed χ^2^ test or Fisher’s exact test, as appropriate. A *p*-value of <0.05 was considered statistically significant. All analyses were performed using validated statistical software.

## 3. Results

### Patient Population

**Figure 1:**
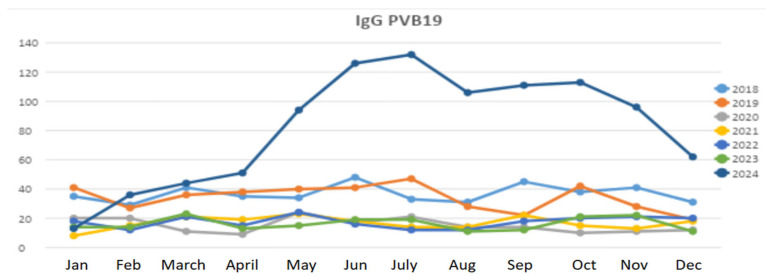
Epidemiological trend of B19V from 2018–2024 in our centre. Lines show the number of pregnant women who were IgG positive for Parvovirus B19 (B19V) in our cohort during this period.

We analysed the overall serological data from 3451 subjects of all ages (mean age = 33 years) examined at our hospital laboratory between 2018 and 2024 (Figure 1). In 2018 and 2019, seroprevalence was comparable to European benchmarks, with rates of 73% and 75%, respectively. However, during 2020–2023, we observed a significant decline in seroprevalence, with an average of approximately 45%, likely reflecting a reduction in testing frequency due to the COVID-19 pandemic. Notably, in 2024, seroprevalence rebounded to 66%, approaching pre-pandemic levels. From a comparison of seroprevalence between the years 2024 and 2018–2019, a statistically significant difference was found (*p* < 0.0001) as well as between 2024 and 2020–2023 (*p* < 0.0001).

**Figure 2:**
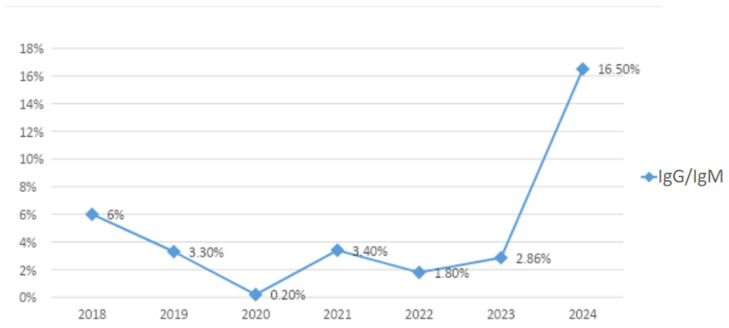
Pregnant women tested in our centre showing both IgM and IgG positivity to B19V from 2018 to 2024. The line shows the percentage of individuals.

Particularly, the epidemiological trend of B19V observed from January to December 2024 showed a progressive increase in B19V cases starting in April 2024, with a peak in July 2024 (Figure 1). Figure 2 illustrates the serological trend of B19V in the general population from 2018 to 2024 in our centre, highlighting a rapid increase in 2024. These time series should be interpreted in the context of our sampling frame: at our centre, the laboratory “general population” is predominantly pregnant women of reproductive age tested during antenatal care (e.g., after relevant exposure, rash illness, or suggestive foetal findings), rather than an age-agnostic community sample. Because B19V testing in pregnancy is targeted (not universal) and IgG seropositivity increases with age, between-year differences may partly reflect changes in obstetric testing volumes and age mix, in addition to genuine shifts in circulation during the 2024 resurgence.

**Figure 3:**
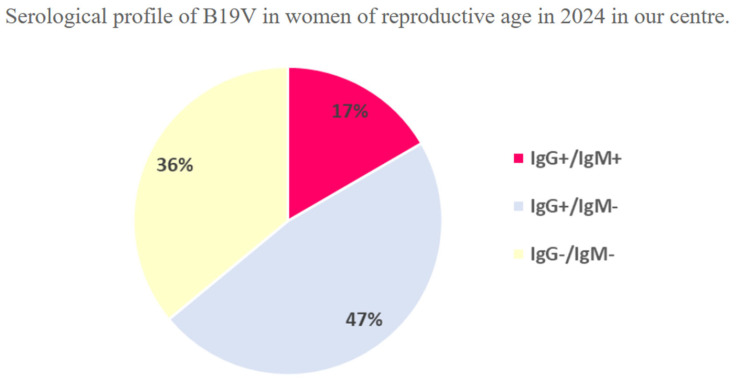
Serological profile of B19V in women of reproductive age in 2024 in our centre.

As shown in Figure 3, in a subgroup analysis of 420 women of reproductive age (mean age = 32.2 years) who underwent serological testing for Parvovirus at our centre in 2024, 17% exhibited both IgG and IgM positivity, consistent with a recent infection, while 47% showed IgG positivity with negative IgM, indicative of a past infection; the remaining 36% were entirely seronegative. Among 51 pregnant women who were both IgG and IgM positive, 37 exhibited primary infection, 12 underwent seroconversion, and 2 had periconceptional infection; delivery outcomes were analysed in a subset of 15 women. The antibody status for Parvovirus was not known for the first group of women, unlike the second group, whose seronegativity was known until the time of counselling.

In our cohort of 70 newborns with suspected congenital B19V infection, the gestational age (GA) at birth ranged from 30.0 to 41.3 weeks, with a mean of 38.81 ± 1.62 weeks, a median of 39.0 weeks, and an interquartile range of approximately 38.1 to 39.2 weeks. Based on the GA classification, 65 neonates (92.9%) were at term (≥37 weeks); 4 neonates (5.7%) were late preterm (34–36 6/7 weeks); only 1 newborn (1.4%) was early preterm (<34 weeks). Out of 70 neonates, Caesarean delivery was performed in 34 cases (48.6%), while vaginal delivery occurred in 36 cases (51.4%). Regarding the birth weight, it ranged from 2420 g to 4490 g, with a mean of 3193 (SD = 388 g), a median of 3185 g, and an interquartile range of approximately 2940 to 3420 g. Our protocol mandated hospitalization for all newborns affected by symptomatic congenital B19V infection; therefore, only seven were admitted and further described in this study, and subsequent protocol revisions favoured outpatient follow-up over routine hospitalization.

Among the seven hospitalised neonates, the duration of hospital stay ranged from 6 to 21 days, with a mean of 12.43 days, a median of 14 days, a standard deviation of 5.56 days, and an interquartile range of 9 days.

Echocardiographic evaluations were normal in all cases. Similarly, ECG recordings revealed no ST-segment abnormalities, and both QT and QTc intervals remained within normal limits, with the exception of one neonate who exhibited arrhythmia.

**Figure 4:**
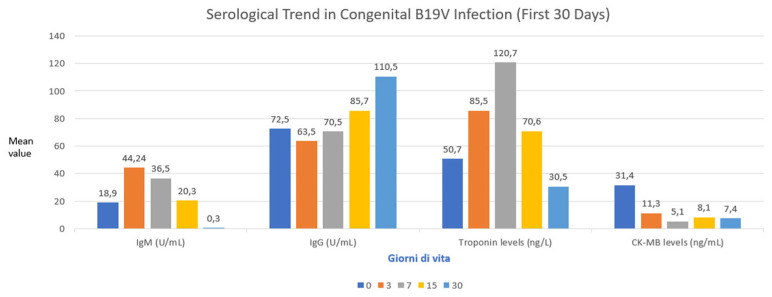
Serological trend in congenital B19V infection (first 30 days).

Figure 4 illustrated the serological trend in congenital B19V infection in our cohort.

**Figure 5:**
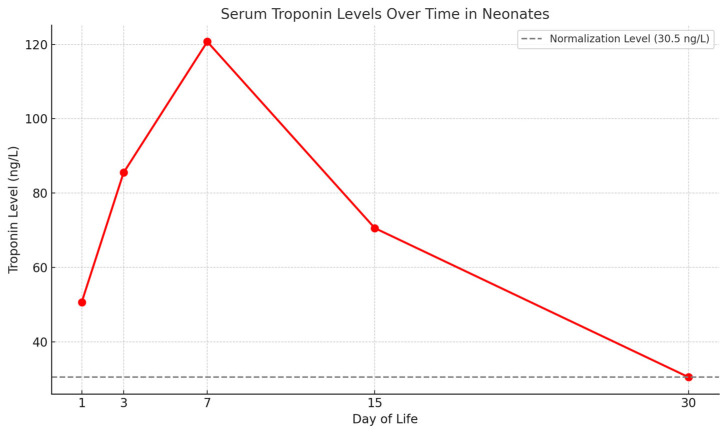
Mean serum troponin levels over days of life in our cohort. The ANOVA test showed *p* value < 0.05. Normal values of troponin, defined as levels ≤20 ng/L, were achieved at 30 days of life in our cohort.

Mean serum troponin levels were 50.7 ng/L on day 1, increased to 85.5 ng/L on day 3, peaked at 120.7 ng/L on day 7, and subsequently decreased to 70.6 ng/L on day 15, eventually normalizing to 30.5 ng/L by approximately one month of age (Figure 4). As shown in Figure 5, mean serum troponin levels exhibited a statistically significant temporal variation over the neonatal period (the ANOVA test showed *p* value < 0.05). Other cardiac biomarkers remained within normal ranges throughout the observation period. Among the neonates tested, CK-MB levels varied significantly. Notably, one patient exhibited a markedly elevated CK-MB level of 65.1 ng/mL, substantially exceeding the upper reference limit of 5.2 ng/mL, suggesting possible myocardial injury. The mean CK-MB value at the first test was 30.45 ng/mL, ranging from 8.4 to 65.1 ng/mL. The dosage was repeated four times until a negative result was achieved.

Serological testing showed that one infant was positive for B19V IgM and negative for IgG, consistent with a recent primary infection due to peri-partum infection. Five children were negative for IgM but positive for IgG, suggesting maternal antibody transfer. One infant showed inconclusive serological results, with borderline IgM and negative IgG.

In our cohort of neonates diagnosed with congenital B19V infection, we evaluated the presence of viral DNA in various biological samples to assess the extent of viremia and potential viral shedding. Among the neonates tested, B19V DNA was detected in the serum of two out of five infants (40%) with a viral load of 10^4^ genome equivalents per millilitre. Another neonate exhibited a high viral load exceeding 10^7^ genome equivalents per millilitre, indicating significant viremia on day 1, which decreased to 4 × 10^5^ on day 3 and 4 × 10^4^ on day 7, maintaining the same viral load for up to 15 days. Comparing the troponin values with those of the viral load in this newborn, it can be seen that the stabilization of the viral load is followed by stabilization of the troponin. Another three neonates had detectable B19V DNA levels below the quantification threshold. All tested samples were negative for B19V DNA, suggesting limited or no viral shedding in these fluids during the neonatal period. The detection of B19V DNA in neonatal serum samples underscores the utility of PCR testing in confirming congenital infection. The presence of high viral loads in some neonates aligns with findings from previous studies, which have reported that B19V DNA can be detected in neonatal blood samples following intrauterine infection. However, the absence of viral DNA in saliva and urine samples suggests that these specimens may have limited diagnostic value for B19V detection in neonates. Regarding inflammatory markers, C-reactive Protein (CRP) and Procalcitonin (PCT), CRP values ranged from 0.2 mg/dL to 5.25 mg/dL. The mean CRP level was approximately 3.1 mg/dL, with a standard deviation of 2.0 mg/dL. Elevated CRP levels, defined as values exceeding 1.0 mg/dL, were observed in 60% of the tested neonates, indicating a moderate inflammatory response. PCT measurements showed values ranging from 0.13 ng/mL to 2.52 ng/mL. The mean PCT level was approximately 1.3 ng/mL, with a standard deviation of 0.9 ng/mL. Elevated PCT levels, considered above 0.5 ng/mL, were present in 50% of the neonates tested, suggesting a systemic inflammatory response.

**Figure 6:**
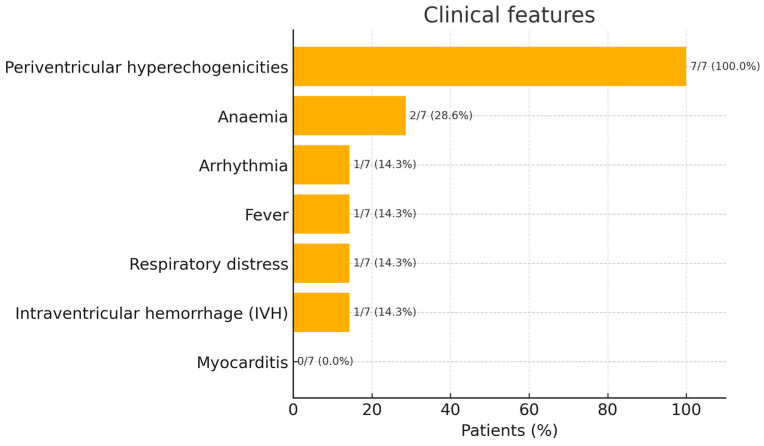
Clinical features in newborns with congenital B19V infection.

The clinical features of our cohort are illustrated in Figure 6. In our cohort of neonates diagnosed with congenital B19V infection, we observed instances of anaemia during the first 30 days of life. Specifically, haemoglobin (Hb) levels as low as 9.8 g/dL and 8.2 g/dL were recorded in two infants. Additionally, cerebral ultrasound examinations revealed multiple periventricular hyperechogenicities in all seven neonates; among these patients, only one presented with IVH. These findings were not attributable to prematurity, suggesting a potential association with B19V infection. One neonate presented with respiratory distress, and another exhibited febrile episodes. Notably, no cases of myocarditis were identified in this cohort. However, one neonate developed a cardiac arrhythmia, underscoring the virus’s potential to affect cardiac conduction systems.

## 4. Discussion

Our prospective cohort documents the re-emergence of congenital B19V in the immediate post-pandemic period and shows that, although biochemical evidence of myocardial stress, systemic inflammation and early cerebral ultrasound abnormalities was frequent, clinically significant cardiorespiratory disease was uncommon and resolved quickly with conservative management. These observations refine the risk profile of infected neonates and underline the importance of tiered laboratory and imaging follow-up rather than systematic admission to intensive care.

The steep rise in maternal and neonatal cases recorded in our centre during 2024 mirrors the pan-European signal reported by national public health agencies and independent laboratories, who describe a three- to five-fold increase after years of suppressed circulation during COVID-19 restrictions [18]. Such oscillations are in keeping with the 3–4-year epidemic cycles historically described for B19V but were accentuated by the immunity gap created when barrier measures limited exposure to common childhood viruses [18].

Our maternal cohort confirms a vertical transmission rate close to one-third, consistent with recent systematic reviews (17–51%) [12]. The high proportion of acute infections (17% of tested women) reinforces the need for heightened vigilance during epidemic waves, particularly as no routine antenatal screening is currently recommended in most guidelines [19].

Peak troponin values on day 7 (median ≈ 120 ng L^−1^) far exceeded sex-specific reference limits, yet echocardiography and ECG remained normal in all but one infant, and biomarker levels normalised by one month. Comparable dissociation between biochemical injury and structural disease has been described in foetal or neonatal case series and experimental models [20]. Collectively, these data suggest that viraemic myocardial damage is mild, patchy and self-limiting. Serial troponin, therefore, appears to be a sensitive, but not specific, marker that can safely guide outpatient follow-up rather than trigger invasive assessment. Troponins are a group of proteins present in skeletal striated muscle tissue and cardiac smooth muscle tissue involved in the regulation of muscle contraction that allows movement. They control the calcium-mediated interaction of actin and myosin (muscle myofibrils) and are classified into three molecular subunits: TnC (troponin C), TnT (troponin T) and TnI (troponin I) [21]. Troponin C binds to calcium ions, troponin T binds to tropomyosin, and troponin I binds to the protein F-actin. While troponin TnC is expressed by both cardiac and skeletal muscle, TnI and TnT are cardiac troponins specific to the heart. These are contractile myofibrillary proteins present exclusively in cardiac myocytes.

During cardiac contraction, the electrical impulse stimulates the release of Ca^2+^ from the sarcoplasmic reticulum, which binds to troponin C (TnC). This binding induces a conformational change in the troponin complex: troponin I (TnI) detaches from actin and tropomyosin moves, exposing binding sites on actin for myosin. Thus, actin–myosin interaction and muscle contraction occur. During relaxation, Ca^2+^ is removed, the troponin complex returns to its resting configuration, and actin–myosin interaction is inhibited [21].

They are released into the blood by the myocardial tissue only following certain events or insults that cause cardiac damage or significant heart suffering. In this case, cardiac muscle cells and myocytes are damaged and die. In fact, in the case of myocardial infarction, ischemia, inflammatory processes, trauma and exposure to toxic agents, cardiac troponins, measurable with a blood chemistry test, increase significantly in the serum [21].

Normal troponin values in newborns are different from those in adults, especially in the first days of life, because they can be influenced by the type of birth (vaginal or caesarean), the prematurity of the newborn, post-natal cardiovascular adaptation and the hypoxic stress of labour. Their maximum value is around 20 ng/L (4), and slightly elevated values at birth can be normal, especially after vaginal birth. However, persistently high or increasing values should raise suspicion of the following: perinatal myocardial distress (asphyxia, hypoxia), congenital heart disease, neonatal sepsis and congenital myocarditis (e.g., from Coxsackie or cytomegalovirus).

In case of infection, there is a release of inflammatory cytokines (TNF-α, IL-1, IL-6, prostaglandins, nitric oxide, oxygen free radicals) and oxidative stress, which directly damage myocardial cells, altering both the cell membrane of cardiomyocytes without complete breakdown of the cells and the mitochondrial function with the consequent release of troponin. Thus, even without obvious necrosis, there is a release of small amounts of troponin due to the sublethal stress of cardiac cells [21,22].

Some studies demonstrate that in inflammatory conditions, cardiac cells can release troponin “reversibly” without complete membrane rupture due to mechanical stress, acidosis, intracellular oedema. This explains why, in many infections, troponin is slightly increased without infarction and, therefore, without electrocardiographic alterations and impairment of cardiac function detectable by echocardiography, which is performed at birth, 7 days, and 1 month with long-term follow up [23,24].

Moderate CRP and PCT elevations were present in roughly half the cohort, echoing the inflammatory profile of larger paediatric datasets [16]. Two infants developed clinically relevant anaemia (Hb ≤ 9 g dL^−1^) within the physiological nadir period, supporting the concept that transient red-cell aplasia can persist post-natally, as repeatedly shown in persistent-viraemia reports [21]. Early full blood count monitoring remains advisable up to eight weeks of age.

Multiple periventricular hyperechogenicities in 11% of our neonates were not attributable to prematurity. Although their prognostic weight is uncertain, they justify scheduled neurodevelopmental assessment, given the 10–13% rate of long-term impairments reported in meta-analyses [11].

Serum PCR detected high-level viraemia in 40% of tested neonates, mirroring the >10^8^ IU mL^−1^ loads reported in neonatal case reports [21]. Saliva and urine were invariably negative, confirming that blood remains the specimen of choice for neonatal diagnosis and that the horizontal transmission risk from infected neonates is probably negligible.

The combination of early troponin surveillance, cranial ultrasound at 1–2 weeks and selective serum PCR appears sufficient to stratify risk without systematic NICU admission, a strategy that reduced in-house stays in our unit from routine admission to only 10% of cases. Additionally, targeted serological testing during peaks, coupled with weekly Doppler surveillance of foetuses with proven maternal infection, remains the most evidence-based approach to prevent hydrops and optimise the timing of intra-uterine transfusion [19]. Finally, our data underscore the need for mandatory reporting of B19V in pregnancy to anticipate epidemic waves and allocate resources for foetal therapy. Regarding the limitations of our study, this was a single-centre study; therefore, the principal limit was the modest sample size. Anyway, our strengths include the prospective design, uniform biomarker timelines and multidisciplinary approach.

Large multicentre registries should (I) define troponin cut-offs predictive of adverse outcomes; (II) characterise the natural history of periventricular hyperechogenicity; (III) explore maternal immunoprophylaxis strategies, including hyper-immune globulin, which remain controversial [12]; and (IV) model the cost-effectiveness of episodic versus universal antenatal screening during epidemic years.

## 5. Conclusions

There is currently no recommendation for the universal screening of pregnant women for B19V infection during prenatal care. However, foetal identification of infection has been proven to be a key strategy in optimizing perinatal outcomes. Congenital B19V infection during the 2024 resurgence was associated with brisk but reversible myocardial enzyme release, moderate systemic inflammation and early neuroimaging changes without overt myocarditis. Elevated troponin levels in neonates with congenital B19V infection do not necessarily indicate structural myocardial damage, as confirmed by normal echocardiographic and electrocardiographic findings. In our study, initially high troponin levels normalised over time, suggesting that while troponin monitoring may be useful for assessing myocardial injury, further research is needed to clarify its relationship with clinical myocardial compromise. The follow-up protocol proposed by our centre, encompassing laboratory, clinical, and instrumental assessments over the first 12 months of life, aims to detect early sequelae and limit organ damage, which, although rare, remains severe and difficult to manage.

## Data Availability

The data presented in this study are available on request from the corresponding author.

## Abbreviations

The following abbreviations are used in this manuscript:

B19V	Parvovirus B19
CK-MB	Creatine Kinase–Myocardial Band
CRP	C-reactive Protein
DNA	Deoxyribonucleic Acid
ECG	Electrocardiogram
GA	Gestational Age
gEq/mL	Genome Equivalents per Millilitre
Hb	Haemoglobin
IgG	Immunoglobulin G
IgM	Immunoglobulin M
IVH	Intraventricular Haemorrhage
LMP	Last Menstrual Period
NICU	Neonatal Intensive Care Unit
PCR	Polymerase Chain Reaction
PCT	Procalcitonin
US	Ultrasound
